# Prostate cancer-specific survival among warfarin users in the Finnish Randomized Study of Screening for Prostate Cancer

**DOI:** 10.1186/s12885-017-3579-8

**Published:** 2017-08-29

**Authors:** Pete T. T. Kinnunen, Teemu J. Murtola, Kirsi Talala, Kimmo Taari, Teuvo L. J. Tammela, Anssi Auvinen

**Affiliations:** 1 University of Tampere, Faculty of Medicine and Life Sciences, Tampere, Finland; 2 Department of Urology, Tampere University Hospital, Tampere, Finland; 3 Finnish Cancer Registry, Helsinki, Finland; 4 Department of Urology, University of Helsinki and Helsinki University Hospital, Helsinki, Finland; 5 University of Tampere, Faculty of Social Sciences, Tampere, Finland

**Keywords:** Prostate cancer, Warfarin, Anticoagulant, Survival, Cohort

## Abstract

**Background:**

Venous thromboembolic events (VTE) are common in cancer patients and associated with higher mortality. In vivo thrombosis and anticoagulation might be involved in tumor growth and progression. We studied the association of warfarin and other anticoagulant use as antithrombotic medication and prostate cancer (PCa) death in men with the disease.

**Methods:**

The study included 6,537 men diagnosed with PCa during 1995-2009. Information on anticoagulant use was obtained from a national reimbursement registry. Cox regression with adjustment for age, PCa risk group, primary therapy and use of other medication was performed to compare risk of PCa death between warfarin users with 1) men using other types of anticoagulants and 2) non-users of anticoagulants. Medication use was analyzed as a time-dependent variable to minimize immortal time bias.

**Results:**

In total, 728 men died from PCa during a median follow-up of 9 years. Compared to anticoagulant non-users, post-diagnostic use of warfarin was associated with an increased risk of PCa death (overall HR 1.47, 95% CI 1.13-1.93). However, this was limited to low-dose, low-intensity use. Otherwise, the risk was similar to anticoagulant non-users. Additionally, we found no risk difference between warfarin and other types of anticoagulants. Pre-diagnostic use of warfarin was not associated with the risk of PCa death.

**Conclusions:**

We found no reduction in risk of PCa death associated with warfarin use. Conversely, the risk was increased in short-term use, which is probably explained by a higher risk of thrombotic events prompting warfarin use in patients with terminal PCa.

**Electronic supplementary material:**

The online version of this article (10.1186/s12885-017-3579-8) contains supplementary material, which is available to authorized users.

## Background

Venous thromboembolism (VTE) has been proposed as prognostic factor in prostate cancer (PCa). VTE is common in cancer patients and associated with poor prognosis, risk of death is 8-fold higher in cancer patient with VTE [1–3]. This applies to PCa as well [4]*.* VTE in PCa patients has been associated with more than 6-fold mortality in both symptomatic and incidental venous thromboembolic diseases [5]. Thus*,* anticoagulant drugs could have an impact on PCa prognosis by reducing deaths from VTE*.* Furthermore, anticoagulants, including warfarin, have shown promising antitumor properties in vivo [6–10] mainly in lung and breast cancer.

Previous fairly small studies on anticoagulant use and PCa survival have reported differing results [11–13]. A potential explanation could be confounding by indication; patients with an advanced cancer are at increased risk for VTE [14], thus more often prescribed anticoagulants compared to people without cancer. We conducted a retrospective, population-based cohort study to assess the association of pre- and post-diagnostic use of warfarin and other anticoagulants with PCa survival in the Finnish Randomized Study of Screening for Prostate Cancer (FinRSPC) [15].

## Methods

### Study cohort

FinRSPC includes 80,458 men aged 55-67 years at baseline (i.e. at FinRSPC randomization). After exclusion of prevalent PCa cases, the men were randomized during 1996-1999 either to PSA screening at four-year intervals (the screening arm) or to no intervention (the control arm). All men were followed via Finnish Cancer Registry, which covers 99% of cancers diagnosed in Finland [16]. This study included 6,537 incident PCa cases diagnosed during 1996-2013. Clinical information included Gleason grade and TNM stage (available for 97.3% and 97.7% of the cases, respectively).

PCa cases were stratified into low/intermediate-risk and high-risk groups according to the definition of the European Association of Urology (EAU) [17]. All M1 cases were included in the high-risk group.

Information on deaths was obtained from the Statistics Finland, which assigns official causes of death based on mandatory death certificates, covering all deaths in Finland [18]. The accuracy of information on PCa deaths was ascertained by the FinRSPC cause of death committee with an excellent concordance between official causes of death and the cause of death committee assignments for PCa (kappa 0.95) [15]. Only deaths with PCa (ICD-10 code C61) as the primary cause of death were regarded as PCa deaths.

A sensitivity analysis with PCa as an intermediate cause of death was also performed.

### Information on anticoagulant usage

In order to obtain information on anticoagulant drug purchases during 1995-2009, the study cohort was linked to a national medication reimbursement database maintained by the Finnish Social Insurance Institution (SII) using the unique personal identification code as the key. As a part of the national health insurance that covers all Finnish citizens, SII provides reimbursements for purchases of physician-prescribed drugs [19].The reimbursement is 50-100 % depending on the indication and severity of the condition. In Finland, anticoagulant drugs are available only through physicians’ prescription, thus all anticoagulant purchases in outpatient setting are registered by the database. Drugs used during hospital inpatient periods are not registered.

All 13 anticoagulant drugs used in outpatient setting during the study period were identified based on their ATC codes (Additional file 1: Table S1). Additionally, we obtained information on cholesterol-lowering drugs, antidiabetic and antihypertensive drugs, aspirin and other NSAIDs and alpha-blockers. We also collected information on primary therapy of PCa (radical prostatectomy, external beam radiation therapy (EBRT), hormonal therapy or active surveillance/watchful waiting). Other medication served as a proxy for co-morbidities, as they may influence survival [20–25].

The information from the National Care Register for Health Care maintained by the National Institute for Health and Welfare included provided diagnoses of conditions serving as indications for anticoagulant use: atrial fibrillation (ICD-10: I48), venous thromboembolism (all recorded I82 diagnoses in the study population), pulmonary embolism (ICD-10: I26.0, I26.9) and thrombocytosis. The register covers all of Finland and registers all diagnoses made during in- and out-patient hospital visits during 1996-2014, but does not cover diagnoses from primary care [26]. Additionally, we stratified the analysis by Charlson Comorbidity Score [27] calculated on basis of the registered diagnoses for subgroup analysis.

### Statistical analysis

Cox proportional hazards regression was used to calculate hazard ratios (HR) and 95% confidence intervals (CIs) for PCa death. Follow-up started at PCa diagnosis and continued until death, emigration from Finland or January 1^st^, 2015, whichever was first. Time metric was years and months since the PCa diagnosis.

We used two different model adjustments. The first Cox regression model was adjusted for age only and the second for age, EAU tumor risk group and other medications. The analysis was performed separately for pre-diagnostic and post-diagnostic use of warfarin and additionally for other types of anticoagulants. Separate comparisons were performed between all anticoagulant users and non-users to estimate the overall effect of anticoagulant usage, and between warfarin users and men using non-warfarin anticoagulants to estimate specific effects of warfarin with simultaneous control of confounding by indication.

For each man in the study cohort, the total annual amount of medication purchases was calculated for each calendar year, and then separately for pre- and post-diagnostic use. Standardization of doses between different anticoagulants was performed by dividing the annual total milligram amount with the standard Defined Daily Dose (DDD) as listed by the WHO [28]. Each year with registered anticoagulant purchases, regardless of the amount, was considered a year of usage. The intensity of the use was calculated by dividing the cumulative annual doses with the number of years of usage.

Pre-diagnostic medication use was analyzed as a time-independent variable, and cumulative pre-diagnostic anticoagulant usage beginning from 1995 was stratified by tertiles. Post-diagnostic use was analyzed as a time-dependent variable; the usage status and cumulative use were updated separately for each year of follow-up, beginning from the year of diagnosis.

All men were categorized as non-users until the potential first anticoagulant purchase. After the first purchase, the status was changed into a user, which was maintained for each year with recorded purchases. Men who discontinued the purchases during the follow-up were kept as ever-users. In analysis of warfarin users compared to non-warfarin users, men were categorized as warfarin users each year with recorded warfarin purchases, even if they had used other types of anticoagulants. Only for years with recorded anticoagulant purchases without warfarin use, were they recorded as non-warfarin users.

Long-term impact of timing of post-diagnostic anticoagulant use was evaluated in lag-time analysis, where anticoagulant exposure was lagged 1 to 3 years forward from the actual year of usage i.e. its effect was ignored for that duration from the first exposure.

Competing risks analysis using Fine and Gray regression method with non-PCa death and major thromboembolic diseases as the competing cause of death was performed.

All statistical analyses were carried out using IBM SPSS Statistics 22.0. All statistical tests are two-sided.

## Results

### Population characteristics

During the median follow-up of 9 years after PCa diagnosis 2,296 men died, of whom 728 from PCa (Table 1). The median follow-up from the diagnosis to PCa death was 3.9 years among men with no post-diagnostic anticoagulant use, 4.8 years among warfarin users and 5.8 years in users of other anticoagulants. High-grade cancers were almost equally distributed in these subcategories. Distribution of background characteristics are presented in Table 1.

**Table 1:** Population characteristis of the study population according to pre- and post-diagnostic anticoagulant usage status

		Pre-diagnostic status			Post-diagnostic status		
		No anticoagulation	Warfarin	Other anticoagulants	No anticoagulation	Warfarin	Other anticoagulants
n of men in the study population		5601	570	366	4485	1074	978
Mean age at diagnosis		67	70	70	67	68	67
PCa deaths		624 (11.1%)	65 (11.4%)	39 (10.7%)	509 (11.3%)	121 (11.3%)	98 (10.0%)
	Median age at death (years)	73	76**	76**	72	75**	74**
Median follow-up from diagnosis to PCa death (years)		4.5	4.5	4.5	3.9	4.8	5.8
EAU prostate cancer risk-group^b^							
	High-grade	1661 (29.6%)	181 (31.8%)	109 (29.8%)	1353 (30.2%)	330 (30.7%)	268 (27.4%)
	Low-/Intermediate-grade	3940 (70.3%)	389 (68.2%)	257 (70.2%)	3132 (69.8%)	744 (69.3%)	710 (72.6%)
Primary therapy^a^							
	Radical prostatectomy	1575 (28.1%)	38 (6.7%)**	39 (10.7%)**	1141 (25.4%)	203 (18.9%)**	308 (31.5%)**
	EBRT	2019 (36.0%)	255 (44.7%)	149 (40.7%)	1647 (36.7%)	444 (41.3%)	332 (33.9%)
	Hormonal treatment	2197 (39.2%)	303 (53.2%)**	169 (46.2%)**	1814 (40.4%)	490 (45.6%)**	365 (37.3%)**
	Active surveillance or watchful waiting	957 (17.1%)	113 (19,8%)	82 (22.4%)	796 (17.7%)	195 (18,2%)	161 (16.5%)
Use of other medication							
	Statin users	2443 (43.6%)	346 (60.7%)**	270 (73.8%)**	1825 (40.7%)	607 (56.5%)**	627 (64.1%)**
	Anti-diabetic drug users	1024 (18.3%)	158 (27.7%)**	92 (25.1%)	780 (17.4%)	277 (25.8%)**	217 (22.2%)**
	Anti-hypertensive drug users	3868 (69.1%)	540 (94.7%)**	340 (92.9%)**	2966 (66.1%)	998 (92.9%)**	784 (80.2%)**
	NSAID users	4806 (85.8%)	487 (85.4%)	343 (93.7%)**	3800 (84.7%)	935 (87.1%)	901 (92.1%)**
	Alpha-blocker users	2509 (44.8%)	320 (56.1%)**	203 (55.5%)**	2017 (45.0%)	536 (49.9%)	479 (49.0%)
	Aspirin users	623 (11.1%)	91 (16.0%)	174 (47.5%)**	393 (8.8%)	151 (14.1%)**	344 (35.2%)**
Recorded diagnoses of:							
	Atrial fibrillation	404 (7.2%)	303 (53.2%)**	27 (7.4%)	129 (2.9%)	571 (53.2%)**	34 (3.5%)
	Thrombotic factors*	198 (3.5%)	93 (16.3%)**	36 (9.8%)	88 (2.0%)	169 (15.7%)**	70 (7.2%)**
Charlson Comorbidity Score							
	0	3469 (61.9%)	226 (39.6%)**	127 (34.7%)**	2955 (65.9%)	449 (41.8%)**	418 (42.7%)**
	1	1093 (19.5%)	147 (25.8%)	113 (30.9%)	822 (18.3%)	272 (25.3%)**	259 (26.5%)**
	2	1039 (18.6%)	197 (34.6%)**	126 (34.4%)**	708 (15.8%)	353 (32.9%)**	301 (30.8 %)**

** *P* for difference compared to non-users < 0.001. Calculated with Chi-square test

^a^Primary treatment modalities not mutually exclusive

^b^Categorized according to criteria of the European Association of Urology (EAU) as of 2015

### Risk of PCa death by pre-diagnostic use of warfarin and other anticoagulants

In general, when compared to anticoagulant non-users, there was no clear association between pre-diagnostic use of warfarin and PCa death in either age-adjusted or multivariable-adjusted analysis (multivariable-adjusted HR 1.15, 95% CI 0.88-1.49). No statistically significant risk trends by cumulative amount or duration of use were observed (Table 2). However, pre-diagnostic use of warfarin for 5 years or more was associated with a borderline significant risk increase in the multivariable-adjusted model (HR 1.49, 95% CI 0.97-2.28).

**Table 2:** Pre-diagnostic use of warfarin compared to anticoagulant non-users and warfarin usage in comparison with other anticoagulant drugs stratified by Defined Daily Doses (DDD), duration and intensity of usage

	n of deaths	Age-adjusted	Multivariable-adjusted
Warfarin compared to non-users			
None	624	Ref	Ref
Any	65	1.11 (0.85-1.44)	1.15 (0.88-1.49)
Amount of warfarin use			
≤200 DDD	24	0.98 (0.65-1.48)	0.97 (0.64-1.47)
201-796 DDD	18	1.09 (0.68-1.74)	1.19 (0.74-1.91)
>796 DDD	23	1.31 (0.86-1.99)	1.37 (0.90-2.09)
Duration of warfarin use			
≤1 year	20	0.91 (0.53-1.56)	0.72 (0.42-1.24)
2-4 years	22	0.96 (0.57-1.61)	0.87 (0.52-1.47)
5 or more years	23	1.33 (0.80-2.23)	1.16 (0.69-1.94)
Intensity of warfarin use			
≤114 DDD/year	21	0.95 (0.56-1.62)	0.79 (0.47-1.35)
115-200 DDD/year	24	1.09 (0.65-1.81)	0.94 (0.57-1.57)
>200 DDD/year	20	1.10 (0.64-1.89)	0.96 (0.56-1.65)

Age-adjusted and multivariable-adjusted hazard ratios (95% CI) related to all PCa deaths

When users of other anticoagulants were used as the reference group, warfarin use was not associated with PCa death. No risk trends by cumulative use were observer either (Table 2).

### Risk of PCa death by post-diagnostic anticoagulant use

In the analysis of post-diagnostic use of warfarin, the overall risk of PCa death was significantly higher among warfarin users in comparison to anticoagulant non-users (multivariable-adjusted HR 1.47, 95% CI 1.13-1.93) (Table 3). The risk association was strongest in low-dose usage (<200 DDD) (multivariable-adjusted HR 2.50, 95% CI 1.86-3.36). Short-term (1 year or less)/low-intensity use (<128 DDD/year) of warfarin was similarly associated with an increased risk of PCa death. As the cumulative amounts increased and the duration of usage extended to 2 years or more, the risk increase was attenuated and was no longer statistically significant.

**Table 3:** Post-diagnostic use of warfarin compared to anticoagulant non-users and seperately to users of other types of anticoagulants stratified by Defined Daily Doses (DDD), duration and intensity of usage

	n of deaths	Age-adjusted	Multivariable-adjusted	1-year lag-time	2-year lag-time	3-year lag-time
Warfarin compared to non-users						
None	509	Ref	Ref	Ref	Ref	Ref
Any	121	**1.46 (1.12-1.90)**	**1.47 (1.13-1.93)**	1.12 (0.85-1.48)	1.12 (0.85-1.48)	1.08 (0.83-1.41)
Amount of warfarin use						
≤200 DDD	63	**2.47 (1.84-3.31)**	**2.50 (1.86-3.36)**	**1.45 (1.04-2.02)**	1.34 (0.99-1.83)	1.26 (0.94-1.70)
200-667 DDD	32	0.87 (0.53-1.42)	0.88 (0.54-1.46)	0.85 (0.52-1.41)	0.76 (0.44-1.32)	0.76 (0.43-1.36)
>667 DDD	26	0.97 (0.56-1.68)	1.02 (0.59-1.78)	1.05 (0.60-1.83)	1.15 (0.67-1.97)	1.04 (0.58-1.85)
Duration of warfarin use						
≤1 year	56	**2.03 (1.47-2.81)**	**2.04 (1.47-2.83)**	1.26 (0.89-1.78)	1.25 (0.91-1.71)	1.15 (0.85-1.57)
2-4 years	44	1.28 (0.88-1.87)	1.30 (0.89-1.90)	1.11 (0.73-1.69)	0.87 (0.53-1.42)	0.93 (0.56-1.54)
5 or more years	21	0.95 (0.49-1.85)	1.05 (0.54-2.04)	1.06 (0.56-1.99)	1.37 (0.76-2.47)	1.22 (0.66-2.26)
Intensity of warfarin use						
≤128 DDD/year	45	**1.92 (1.34-2.74)**	**1.91 (1.34-2.74)**	1.31 (0.91-1.88)	1.35 (0.98-1.86)	1.21 (0.89-1.66)
128-200 DDD/year	51	**1.74 (1.24-2.45)**	**1.77 (1.25-2.50)**	1.13 (0.74-1.74)	0.93 (0.57-1.51)	0.94 (0.57-1.56)
>200 DDD/year	25	0.73 (0.40-1.33)	0.78 (0.43-1.43)	0.99 (0.58-1.69)	0.97 (0.56-1.69)	0.99 (0.56-1.77)
Warfarin compared to other anticoagulant drugs						
Non-warfarin anticoagulant users	98	Ref	Ref	Ref	Ref	Ref
Warfarin users	121	1.13 (0.79-1.61)	1.01 (0.71-1.44)	0.93 (0.64-1.35)	1.02 (0.70-1.48)	0.93 (0.65-1.33)
Amount of warfarin use						
≤200 DDD	63	**1.85 (1.25-2.74)**	**1.63 (1.10-2.42)**	1.15 (0.75-1.77)	1.19 (0.78-1.80)	1.06 (0.71-1.58)
200-667 DDD	32	0.65 (0.37-1.14)	0.58 (0.33-1.01)	0.68 (0.38-1.20)	0.67 (0.36-1.25)	0.64 (0.34-1.21)
>667 DDD	26	0.72 (0.39-1.33)	0.67 (0.36-1.23)	0.83 (0.45-1.55)	1.02 (0.56-1.87)	0.87 (0.46-1.64)
Duration of warfarin use						
≤1 year	56	1.52 (1.00-2.31)	1.33 (0.88-2.02)	1.00 (0.64-1.55)	1.11 (0.73-1.68)	0.97 (0.65-1.45)
2-4 years	44	0.96 (0.61-1.52)	0.85 (0.54-1.34)	0.88 (0.53-1.46)	0.77 (0.44-1.35)	0.78 (0.44-1.38)
5 or more years	21	0.71 (0.35-1.46)	0.69 (0.34-1.40)	0.84 (0.42-1.67)	1.22 (0.64-2.33)	1.02 (0.53-2.00)
Intensity of warfarin use						
≤128 DDD/year	45	1.44 (0.93-2.24)	1.25 (0.80-1.95)	1.04 (0.66-1.64)	1.20 (0.78-1.83)	1.02 (0.68-1.53)
128-200 DDD/year	51	1.31 (0.85-2.01)	1.16 (0.75-1.78)	0.90 (0.54-1.49)	0.82 (0.47-1.44)	0.79 (0.45-1.40)
>200 DDD/year	25	0.55 (0.29-1.05)	**0.51 (0.27-0.98)**	0.79 (0.43-1.44)	0.86 (0.46-1.60)	0.83 (0.44-1.57)

Age-adjusted, multivariable-adjusted and lag-time hazard ratios (95% CI) related to all PCa deaths. Statistically significant results are bolded

The risk PCa death was similar for warfarin and other anticoagulants (multivariable-adjusted HR 1.01, 95% CI 0.71-1.44). In the stratified analysis, short-term use of warfarin was associated with an increased risk of PCa death, but again the risk increase was smaller in high-intensity use (Table 3).

### Lag-time analysis

In order to evaluate long-term effects of warfarin use and minimize bias due to thrombotic events triggering the prescription, we performed an analysis lagging warfarin use by one, two and three years, i.e. relating it to events at least 1-3 years later than the exposure to allow for latency. The risk increase compared to anticoagulant non-users was decreased already in the analysis lagged by one year, but remained non-significantly elevated (Table 3). The risk increase noted in low-dose usage was found in the 1-year lagged analysis, but after 2-year lag-time it was only borderline significant (two-year lag-time for ≤200 DDD HR 1.34, 95% CI 0.99-1.83). In general, extending the lag-time to two or three years did not substantially alter the results compared to one-year lag-time analysis.

Lag-time analysis of warfarin use relative to usage of other anticoagulant drugs was not substantially altered compared with the non-lagged results. No risk increase among short-term users or decreased risk among high-intensity users was found in the lagged analysis (Table 3).

### Subgroup analyses

**Fig. 1:**
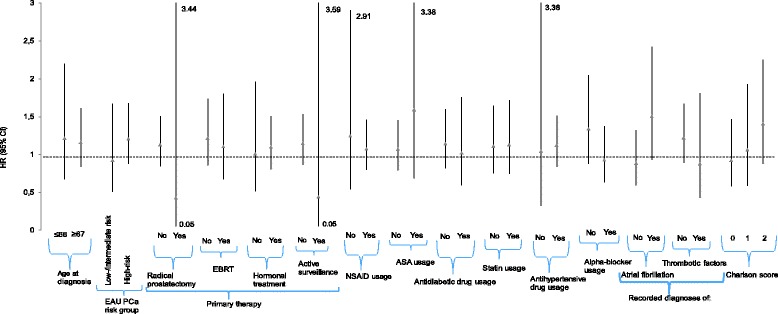
Pre-diagnostic subgroup analysis of warfarin usage compared to anticoagulant non-users

**Fig. 2:**
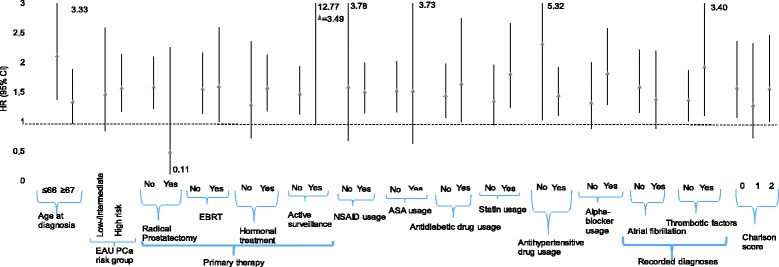
Post-diagnostic subgroup analysis of warfarin usage compared to anticoagulant non-users

No clear risk modification by any background variable was present between pre-diagnostic warfarin use and risk PCa death (Fig. 1). The same applied also to post-diagnostic use and risk of PCa death (Fig. 2). Effect modification was not found when comparing warfarin usage to other types of anticoagulants in pre- and post-diagnostic setting (Additional file 2: Figure S1 and Additional file 3: Figure S2)

### Sensitivity analysis

As part of sensitivity analyses, users of any anticoagulants including warfarin were compared to non-users. There was a borderline significant risk increase among pre-diagnostic users of anticoagulants (multivariable-adjusted HR 1.19, 95% CI 0.96-1.48) (Additional file 4: Table S2).

In analysis of post-diagnostic use, the risk was increased (HR 1.58, 95% CI 1.27-1.97), but no apparent risk trends by cumulative amount or duration were observed. In the lag-time analyses, no statistically significant risk increase was present (Additional file 4: Table S3).

No clear effect modification was observed for pre- nor post-diagnostic use of all anticoagulants (Additional file 5: Figure S3 and Additional file 6: Figure S4).

### Competing risk analysis

In a Fine-Gray regression analysis with non-PCa deaths as competing cause of death, no risk difference was observed between users of warfarin and other anticoagulants (HR 0.95, 95% CI 0.74-1.24). The findings were similar when deaths caused by pulmonary embolism or stroke were analyzed as the competing causes of death (HR 0.95, 95% CI 0.73-1.23).

## Discussion

Short-term and low-dose warfarin use after the diagnosis was associated with increased risk of PCa death compared to anticoagulant non-users. However, in analyses allowing for lag-time, the risk was attenuated after one year and disappeared in 2-year or 3-year lag-time analysis. This suggests that the risk increase occurs only for a short time period after starting drug use. We found no statistically significant risk difference between users of warfarin and other types of anticoagulants. Additionally, we found no association between pre-diagnostic use of warfarin and PCa survival. Although the indication for anticoagulant treatment did not modify the risk association, it is likely that the short-term increased risk is explained by thrombotic events as indications for anticoagulation. In epidemiological literature this phenomenon where pharmaceutical drug is prescribed for early manifestation of yet un-diagnosed disease is referred to as ‘protopathic bias’ [29].

Previous studies on warfarin and PCa mortality are sparse. To date, three published studies have assessed the association between PCa survival and use of vitamin K antagonists [11–13]. Tagalakis et al. found an increased risk of PCa death associated with one-year or ever-use of warfarin in after PCa diagnosis [11]. Our study is consistent with an increased risk associated with short-term use. In contrast to Tagalakis et al., we were able to evaluate cumulative amounts and intensity of use and found that high-dose or long-term use are not associated with risk increase.

In a cohort study including 12,186 men with PCa, but no information on stage no evidence was found for an association between PCa death and pre-diagnostic use of warfarin during a mean follow-up of 3.7 years [12]. In a sensitivity analysis post-diagnostic use was associated with an increased risk of PCa death. Evaluation of cumulative amounts, duration nor intensity of use for post-diagnostic use was not possible. Concordantly, we observed no risk increase for pre-diagnostic warfarin use. Compared to the previous study, we had higher of PCa deaths and were able to evaluate cumulative amount of post-diagnostic use. We found a risk increase only for some subgroups.

A third study of Park et al. [13] was limited to 247 patients with metastatic PCa receiving docetaxel chemotherapy, and included only 17 LMWH users and 12 warfarin users. LMWH was associated with an improved survival, whereas warfarin was not. In our larger and more comprehensive study, we found no evidence for improved survival among men using other types of anticoagulants.

In vitro studies have suggested that the coagulation cascade and thrombocytes might be involved in tumor growth and progression, and that anticoagulant drugs could in theory improve prognosis [6–10]. However, in epidemiologic studies, confounding by indication dilutes this potential effect, as advanced cancer is associated with an increased risk of thrombosis and consequently with likelihood of initiating anticoagulant treatment. We controlled for such bias by comparing users of different types of anticoagulants and performing a competing risk analysis. We found no significant risk difference, which does not support the putative beneficial effect of warfarin or any other anticoagulants.

The main strengths of this study are the large population-based cohort as well as comprehensive and our ability to use detailed, nationally comprehensive register-based information on anticoagulant use unaffected by recall bias. Being able to study large populations through national registries allows us to estimate even relatively uncommonly used drugs such as anticoagulants as cancer risk factor. Our study included substantially more warfarin users than in previous studies. We were able to analyze post-diagnostic use of warfarin more comprehensively than before. We also performed lag-time analyses to estimate the risk associations allowing for latency and removing effects limited to the immediate period following first subscription. Furthermore, we had longer follow-up than in previous studies.

Our study also has some limitations. We were not able to adjust our analysis for smoking, life-style factors or BMI which may be associated with risk of PCa death [30–33]. Nevertheless, we were able to adjust for Charlson Comorbidity Index and this enabled adjustment for co-existing morbidities. Furthermore, our study was not randomized, and is thus prone to residual confounding.

## Conclusion

In a population-based setting, warfarin or other types of anticoagulants are not associated with improved PCa prognosis. Conversely, in short-term use risk of PCa death was increased, which is most likely due to thrombosis caused by an advanced cancer, as the risk increase was not observed in long term.

## Additional files

Additional file 1: Table S1. ATC codes for anticoagulant drugs included in the study. (DOCX 207 kb)

Additional file 2: Figure S1. Pre-diagnostic subgroup analysis between users of warfarin and other anticoagulant drugs. (DOCX 224 kb)

Additional file 3: Figure S2. Post-diagnostic subgroup analysis between users of warfarin and users of other anticoagulant drugs. (DOCX 224 kb)

Additional file 4: Table S2. Pre-diagnostic analysis of combined anticoagulant drug usage stratified by number of Defined Daily Doses (DDD), duration and intensity of usage. Age-adjusted and multivariable-adjusted hazard ratios (95% CI) related to all PCa deaths. **Table S3.** Post-diagnostic results for combined anticoagulant usage. Age-adjusted, multivariable-adjusted and lag-time hazard ratios (95% CI) related to all PCa deaths. (DOCX 405 kb)

Additional file 5: Figure S3. Subgroup analysis of pre-diagnostic combined anticoagulant usage compared to non-users. (DOCX 224 kb)

Additional file 6: Figure S4. Subgroup analysis of combined anticoagulant usage compared to non-users in post-diagnostic setting. (DOCX 224 kb)

## Abbreviations

VTE: Venous thromboembolism
PCa: Prostate cancer
FinRSPC: the Finnish Randomized Study of Screening for Prostate Cancer
EAU: the European Association of Urology
SII: (Finnish) Social Insurance Institution
EBRT: external beam radiation therapy
HR: Hazard ratio
CI: Confidence interval
DDD: Defined Daily Dose
