# Supplementary material for: Prostate cancer-specific survival among warfarin users in the Finnish Randomized Study of Screening for Prostate Cancer
Source: BMC Cancer. 2017 Aug 29;17:585. doi: 10.1186/s12885-017-3579-8 (PMC5575900; doi:10.1186/s12885-017-3579-8)
Supplement: Supplementary file 1 [file 12885_2017_3579_MOESM1_ESM.docx]

Supplementary Table 1

**Supplementary table 1**	ATC codes for anticoa
**Anticoaglant drug**	
Vitamin K antagonists	
	Warfarin
	Fenindion
Heparins	
	Dalteparin
	Enoxaparin
	Tinzaparin
Aggregation Inhibitors	
	Clopidogrel
	Dipyridamole
	Iloprost
	Ticlopidine
