# Supplementary material for: Prostate cancer-specific survival among warfarin users in the Finnish Randomized Study of Screening for Prostate Cancer
Source: BMC Cancer. 2017 Aug 29;17:585. doi: 10.1186/s12885-017-3579-8 (PMC5575900; doi:10.1186/s12885-017-3579-8)
Supplement: Supplementary file 2 [file 12885_2017_3579_MOESM2_ESM.docx]

Supplementary Figure 1 Pre-diagnostic subgroup analysis between users of warfarin and other other anticoagulant drugs

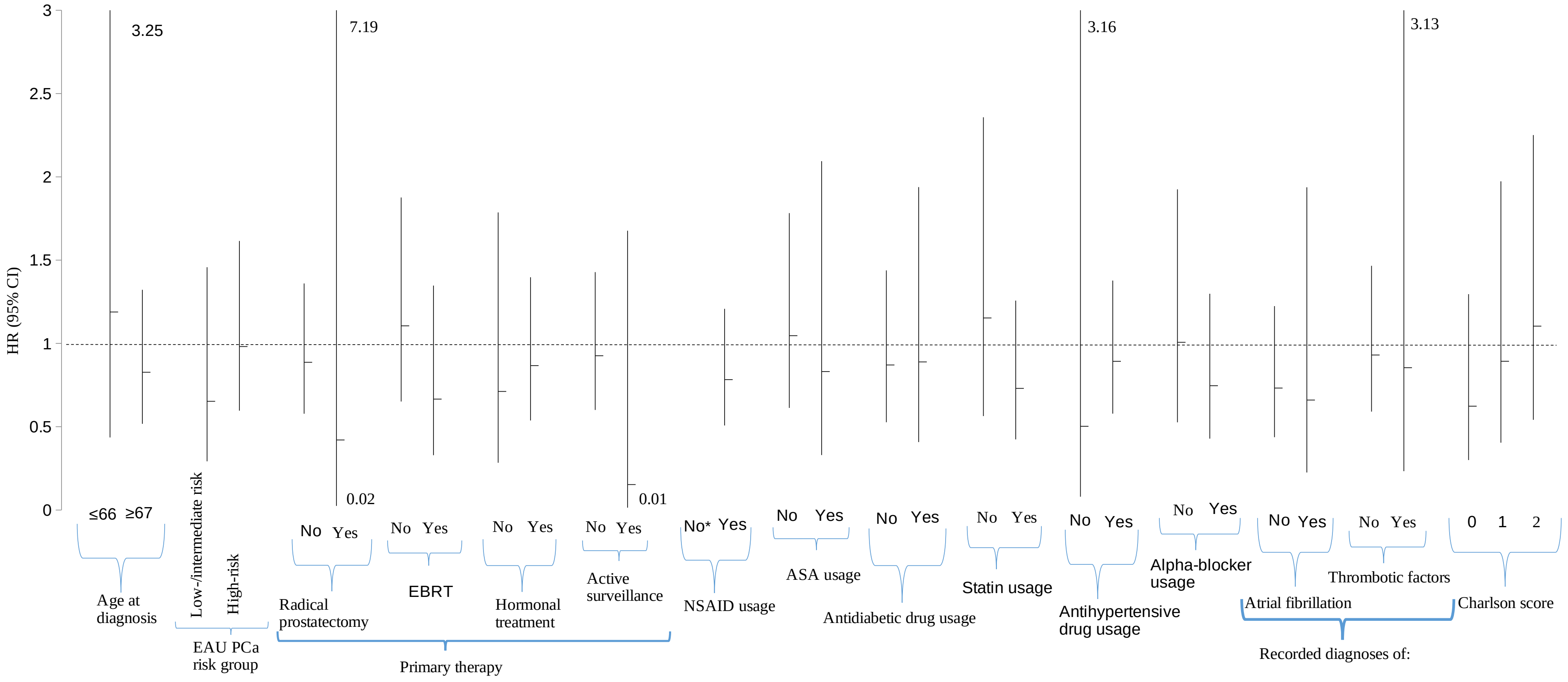

*= Not Applicable
