# Supplementary material for: Prostate cancer-specific survival among warfarin users in the Finnish Randomized Study of Screening for Prostate Cancer
Source: BMC Cancer. 2017 Aug 29;17:585. doi: 10.1186/s12885-017-3579-8 (PMC5575900; doi:10.1186/s12885-017-3579-8)
Supplement: Supplementary file 3 [file 12885_2017_3579_MOESM3_ESM.docx]

Supplementary Figure 2 Post-diagnostic subgroup analysis between users of warfarin and users of other anticoagulant drugs

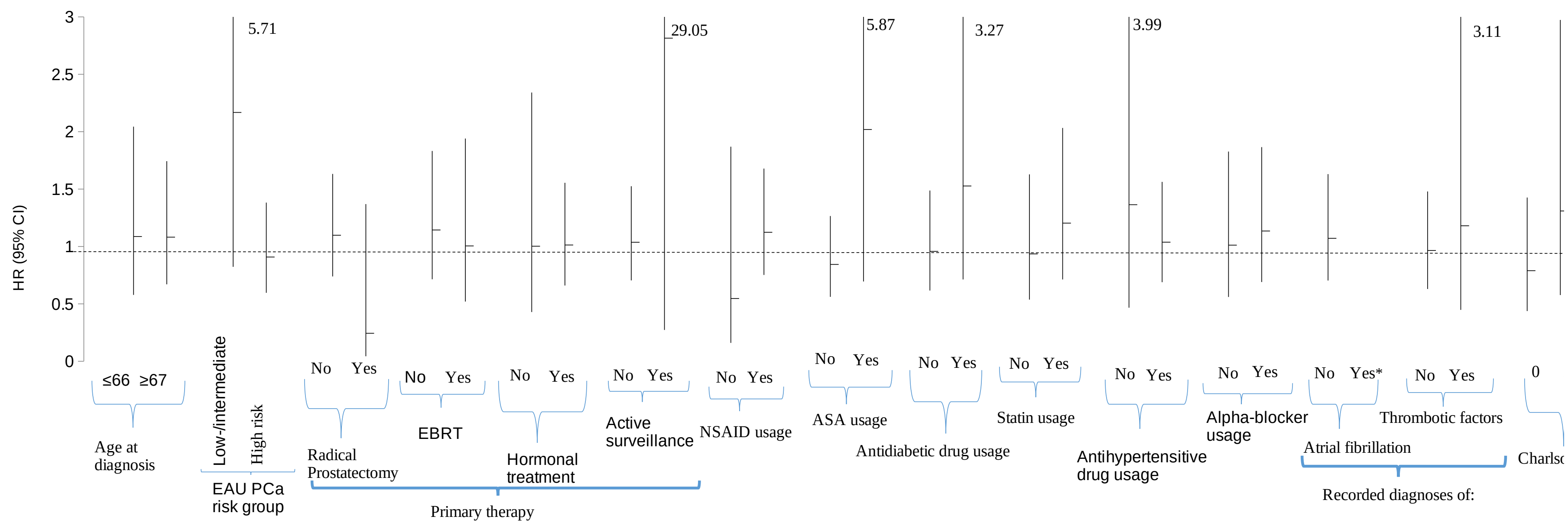

*=Not Applicable
