# Supplementary material for: Prostate cancer-specific survival among warfarin users in the Finnish Randomized Study of Screening for Prostate Cancer
Source: BMC Cancer. 2017 Aug 29;17:585. doi: 10.1186/s12885-017-3579-8 (PMC5575900; doi:10.1186/s12885-017-3579-8)
Supplement: Supplementary file 4 [file 12885_2017_3579_MOESM4_ESM.docx]

Supplementary Table 2 Pre-diagnostic analysis of combined anticoagulant drug usage stra
of Defined Daily Doses (DDD), duration and intensity of usage. Ag[illegible]
multivariable-adjusted hazard ratios (95% CI) related to all PCa de[illegible]

		n of deaths	Age-adjusted
Anticoagulants compared to non-users			
	None	624	Ref
	Any	104	1.09 (0.88-1.35)
Amount of anticoagulant drug use			
	≤133 DDD	37	1.12 (0.80-1.57)
	133-600 DDD	31	0.94 (0.65-1.35)
	>600 DDD	36	1.23 (0.88-1.73)
Duration of anticoagulant drug use			
	≤1 year	44	1.11 (0.82-1.51)
	2-3 years	29	1.06 (0.73-1.54)
	4 or more years	31	1.09 (0.76-1.58)
Intensity of anticoagulant drug use			
	≤84 DDD/year	38	1.09 (0.78-1.52)
	84-200 DDD	39	1.16 (0.84-1.61)

Supplementary Table 3 Post-diagnostic results for combined anticoagulant usage. Age-adjusted, multivariable-adjusted and lag-time hazard ratios (95% CI) rela all PCa deaths. Statistically significant results are bolded.

		n of deaths	Age-adjusted	Multivariable-adjusted	1-year lag-time	2-year lag-time	3-year lag-ti
Anticoagulants compared to non-users							
	None	509	Ref	Ref	Ref	Ref	Ref
	Any	219	**1.52 (1.23-1.88)**	**1.58 (1.27-1.97)**	1.22 (0.97-1.53)	1.05 (0.83-1.32)	1.09 (0.88-1
Amount of anticoagulant drug use							
	≤120 DDD	66	1.38 (0.99-1.92)	**1.56 (1.11-2.17)**	1.20 (0.88-1.65)	1.21 (0.91-1.59)	1.17 (0.90-1
	120-583 DDD	88	**1.51 (1.15-1.99)**	**1.58 (1.19-2.08)**	1.19 (0.87-1.63)	1.06 (0.75-1.50)	0.87 (0.58-1
	>583 DDD	65	1.37 (0.98-1,92)	**1.47 (1.04-2.07)**	1.24 (0.85-1.82)	1.12 (0.74-1.69)	1.46 (1.00-2
Duration of anticoagulant drug use							
	≤1 year	113	**1.69 (1.32-2.17)**	**1.84 (1.43-2.37)**	1.20 (0.91-1.57)	1.19 (0.93-1.53)	1.11 (0.87-1
	2-3 years	63	1.35 (0.97-1.88)	1.39 (1.00-1.95)	1.31 (0.92-1.87)	1.03 (0.68-1.57)	1.13 (0.75-1
	4 or more years	43	1.01 (0.64-1.58)	1.11 (0.71-1.74)	1.09 (0.69-1.72)	1.11 (0.70-1.76)	1.30 (0.82-2
Intensity of anticoagulant drug use							
