# Supplementary material for: Prostate cancer-specific survival among warfarin users in the Finnish Randomized Study of Screening for Prostate Cancer
Source: BMC Cancer. 2017 Aug 29;17:585. doi: 10.1186/s12885-017-3579-8 (PMC5575900; doi:10.1186/s12885-017-3579-8)

Supplementary Figure 3 Subgroup analysis of pre-diagnostic combined anticoagulant usage compared to non-users

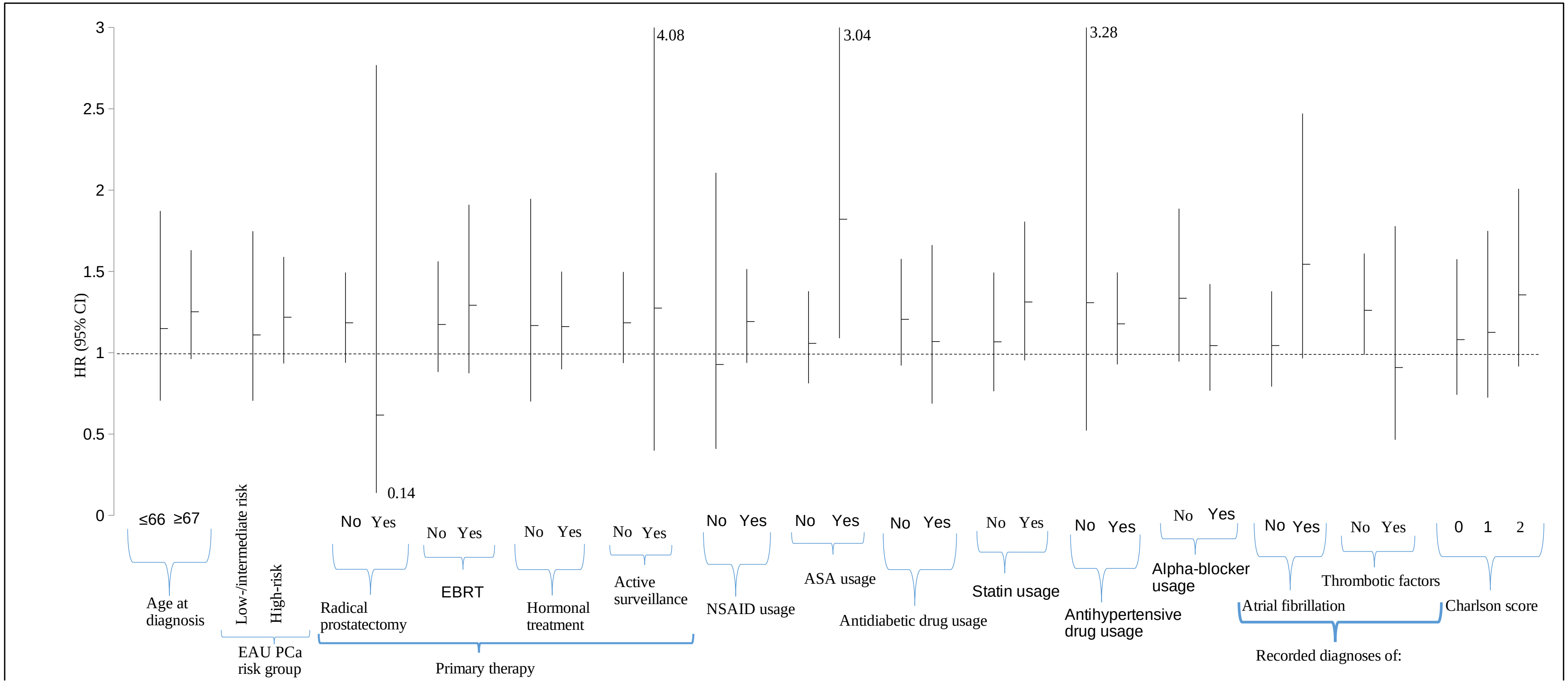

Supplement: Supplementary file 5 [file 12885_2017_3579_MOESM5_ESM.docx]
